# Chikungunya Virus in Macaques, Malaysia

**DOI:** 10.3201/eid2109.150439

**Published:** 2015-09

**Authors:** I-Ching Sam, Chong Long Chua, Jeffrine J. Rovie-Ryan, Jolene Y.L. Fu, Charmaine Tong, Frankie Thomas Sitam, Yoke Fun Chan

**Affiliations:** University Malaya, Kuala Lumpur, Malaysia (I-C. Sam, C.L. Chua, J.Y.L. Fu, C. Tong, Y.F. Chan);; Department of Wildlife and National Parks Peninsular Malaysia, Kuala Lumpur (J.J. Rovie-Ryan, T. Sitam)

**Keywords:** chikungunya virus, viruses, nonhuman primates, macaques, Macaca fascicularis, Macaca, monkeys, neutralizing antibodies, seroprevalence, Malaysia

**To the Editor:** In the past 10 years, chikungunya virus (CHIKV) has caused global epidemics of fever, rash, and arthralgia affecting millions of humans, most recently in the Americas (*1*). CHIKV is an alphavirus transmitted by *Aedes* spp. mosquitoes. This virus has been isolated from wild vertebrates, particularly nonhuman primates (NHPs), in Africa (*2*). This sylvatic cycle might maintain the virus during interepidemic periods. The role of sylvatic cycles in Asia is less clear.

Encroachment of human settlements into forests has caused increased conflict between humans and macaques for space and resources in urban and rural areas. This interface exposes humans to zoonotic pathogens found in monkeys, such as CHIKV, dengue virus, and *Plasmodium knowlesi*. The most common macaque species in Peninsular Malaysia is the long-tailed macaque (*Macaca fascicularis*); an estimated population of >130,000 monkeys live in human-populated areas (*3*). We determined the potential role of long-tailed macaques in conflict with humans as a reservoir of CHIKV in Malaysia.

In response to reports of long-tailed macaques in human-populated areas, the Malaysian Department of Wildlife and National Parks traps monkeys in these areas and relocates them to forest areas. As part of the Wildlife Disease Surveillance Program conducted by Outbreak Response Team of this department, with assistance from the EcoHealth Alliance, serum samples were collected from 147 long-tailed macaques at >20 sites in the states of Selangor (88 monkeys), Negeri Sembilan (21), Perak (18), Pahang (17), and Penang (3) (Figure). Samples were collected in October–November 2009 and October 2010, just after a nationwide outbreak of CHIKV that affected >13,000 persons in 2008–2009 (*4*). These samples represent 0.05%–0.29% of estimated populations of long-tailed macaques in human-populated areas in these 5 states (*3*).

**Figure:**
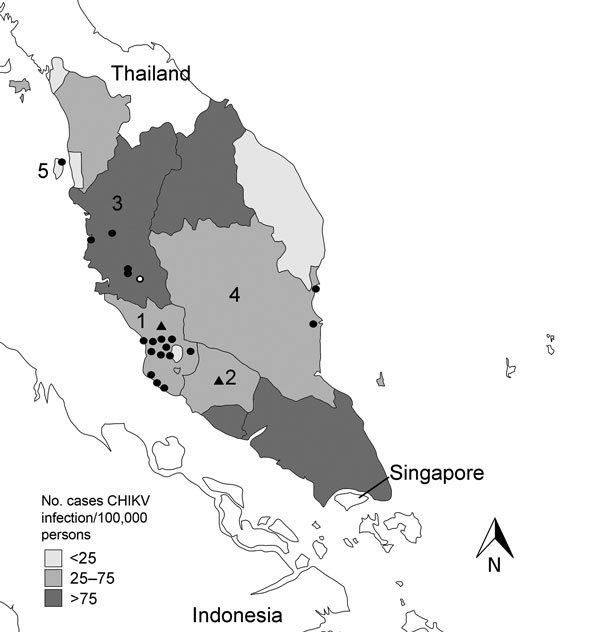
Cases of human infection with chikungunya virus (CHIKV) per 100,000 persons in 5 states in Peninsular Malaysia, 2008–2009, and sites where monkeys were sampled in 2009–2010. Published CHIKV case numbers were used (*4*), and published estimated populations of monkeys in 2011 were reduced by an annual growth rate of 5% to obtain population estimates for 2010 (*3*). Solid circles indicate monkey sampling sites, triangles indicates sites from which samples were obtained (where the specific locations was not known), and open circle indicates site from which a sample was obtained from a seropositive macaque. Numbers indicate states where monkeys were sampled. 1, Selangor, 88 monkeys (0.29%) sampled of an estimated population of 29,924; 2, Negeri Sembilan, 21/10,133 (0.21%); 3, Perak, 18/15,114 (0.12%); 4, Pahang, 17/12,590 (0.14%); 5, Penang, 3/6,019 (0.05%).

A seroneutralization assay was performed by using baby hamster kidney cells to screen for neutralizing antibodies against CHIKV in heat-inactivated monkey serum samples. Samples at a 1:20 dilution that neutralized CHIKV in <2 days were confirmed as positive by using a described immunofluorescence-based cell infection assay (*5*) with modifications. Serially diluted serum samples were mixed with equal volumes of CHIKV suspensions at a multiplicity of infection of 10 and inoculated into baby hamster kidney cells. After incubation for 6 h at 37°C, cells were fixed, processed, and immunostained with a monoclonal antibody. Fluorescence was determined by using the Cellomics High Content Screening ArrayScan VTI imaging system (ThermoFisher Scientific, Waltham, MA, USA).

Despite the recent widespread CHIKV outbreak in humans and proximity of sampled macaques to humans in Malaysia, CHIKV neutralizing antibodies were detected in only 1 (0.7%) of 147 macaques. This seropositive macaque was captured in Kampung Jeram Mengkuang (4.06°N, 101.24°E) in Perak, one of the most affected states during the 2008–2009 outbreak (*4*). All serum samples tested showed negative PCR results for the CHIKV envelope 1 protein gene.

CHIKV neutralizing antibodies have also been detected in NHPs in Thailand (*6*) and Malaysia (*7*). In the study in Malaysia, 6 (1.5%) of 393 long-tailed macaques were seropositive (*7*). A recent study in Mauritius reported neutralizing antibodies in just 1 (0.7%) of 134 long-tailed macaques after a large human outbreak in 2006 (*8*). In another study in Malaysia, 105 wild long-tailed macaques were sampled from several sites in 3 states during 2007–2008; CHIKV was isolated from 4 (3.8%) samples from 1 site (Kuala Lipis in Pahang) (*9*). This site is 90 km from the village where the 1 seropositive monkey was trapped in our study. In addition, a variety of domestic and wild vertebrates, including horses, cattle, pigs, rats, squirrels, bats, and chickens, have been reported to be seropositive for CHIKV (*2**,**6**–**8*).

These results indicate that CHIKV infects long-tailed macaques in Malaysia, but seroprevalence rates are low, and there is little evidence of viremia, except at the 1 specific site in Kuala Lipis. Although experimental infection of long-tailed macaques resulted in detectable CHIKV antigen in macrophages for >3 months, infectious CHIKV is not detectable beyond 44 days (*10*), and long-term neutralizing immunity is present for >180 days (*5*). However, there is no evidence for long-term active CHIKV infection and its recrudescence in macaques or humans.

A limitation of our study was the relatively small number of monkeys sampled. Although we found no overall significant correlation between incidence of human cases of infection with CHIKV and estimated number of long-tailed macaques per 100,000 persons in each state (r^2^ = 0.05, p = 0.49), we cannot exclude the involvement of long-tailed macaques in a local outbreak at a specific site. Long-term dynamics of antibodies against CHIKV in long-tailed macaques are not known, which might affect sensitivity of detection assays.

We conclude that long-tailed macaques in conflict with humans in specific areas probably played a small part in transmission of CHIKV during recent large outbreaks in humans in Malaysia. Human–mosquito–human transmission and travel by infected humans were probably the major factors involved in spread of this virus. If a true sylvatic reservoir that effectively maintains CHIKV is present in Malaysia, long-tailed macaques might play only a minor role. In addition, involvement of other NHPs and mammals remains to be elucidated.

## References

[1] Rougeron V, Sam IC, Caron M, Nkoghe D, Leroy E, Roques P. Chikungunya, a paradigm of neglected tropical disease that emerged to be a new health global risk. J Clin Virol. 2015;64:144–52 . 10.1016/j.jcv.2014.08.032 25453326

[2] Diallo M, Thonnon J, Traore-Lamizana M, Fontenille D. Vectors of chikungunya virus in Senegal: current data and transmission cycles. Am J Trop Med Hyg. 1999;60:281–6 . 10072152 10.4269/ajtmh.1999.60.281

[3] Karuppannan K, Saaban S, Mustapa AR, Zainal Abidin FA, Azimat NA, Keliang CJ. Population status of long-tailed macaque (*Macaca fascicularis*) in Peninsular Malaysia. J Perinatol. 2014;3:2.

[4] Chua KB. Epidemiology of chikungunya in Malaysia: 2006–2009. Med J Malaysia. 2010;65:277–82 . 21901945

[5] Kam YW, Lee WW, Simarmata D, Le Grand R, Tolou H, Merits A, Unique epitopes recognized by antibodies induced in chikungunya virus–infected non-human primates: implications for the study of immunopathology and vaccine development. PLoS ONE. 2014;9:e95647. 10.1371/journal.pone.0095647 24755730 PMC3995782

[6] Halstead SB, Udomsakdi S. Vertebrate hosts of chikungunya virus. Bull World Health Organ. 1966;35:89 . 20604287 PMC2476142

[7] Marchette NJ, Rudnick A, Garcia R, MacVean DW. Alphaviruses in Peninsular Malaysia: I. Virus isolations and animal serology. Southeast Asian J Trop Med Public Health. 1978;9:317–29 . 34888

[8] Vourc’h G, Halos L, Desvars A, Boué F, Pascal M, Lecollinet S, Chikungunya antibodies detected in non-human primates and rats in three Indian Ocean islands after the 2006 ChikV outbreak. Vet Res. 2014;45:52. 10.1186/1297-9716-45-52 24885529 PMC4018978

[9] Apandi Y, Nazni WA, Noor Azleen AZ, Vythilingam I, Noorazian MY, Azahari AH, The first isolation of chikungunya virus from nonhuman primates in Malaysia. Journal of General and Molecular Virology. 2009;1:35–9.

[10] Labadie K, Larcher T, Joubert C, Mannioui A, Delache B, Brochard P, Chikungunya disease in nonhuman primates involves long-term viral persistence in macrophages. J Clin Invest. 2010;120:894–906. 10.1172/JCI40104 20179353 PMC2827953

